# Publisher Correction: FOXO1 is a master regulator of memory programming in CAR T cells

**DOI:** 10.1038/s41586-024-07450-9

**Published:** 2024-04-23

**Authors:** Alexander E. Doan, Katherine P. Mueller, Andy Y. Chen, Geoffrey T. Rouin, Yingshi Chen, Bence Daniel, John Lattin, Martina Markovska, Brett Mozarsky, Jose Arias-Umana, Robert Hapke, In-Young Jung, Alice Wang, Peng Xu, Dorota Klysz, Gabrielle Zuern, Malek Bashti, Patrick J. Quinn, Zhuang Miao, Katalin Sandor, Wenxi Zhang, Gregory M. Chen, Faith Ryu, Meghan Logun, Junior Hall, Kai Tan, Stephan A. Grupp, Susan E. McClory, Caleb A. Lareau, Joseph A. Fraietta, Elena Sotillo, Ansuman T. Satpathy, Crystal L. Mackall, Evan W. Weber

**Affiliations:** 1 Center for Cancer Cell Therapy, Stanford Cancer Institute, Stanford University School of Medicine, Stanford, CA USA; 2 Department of Pediatrics, Perelman School of Medicine, University of Pennsylvania, Philadelphia, PA USA; 3 Center for Cellular and Molecular Therapeutics, Children’s Hospital of Philadelphia, Philadelphia, PA USA; 4 Center for Cellular Immunotherapies, Perelman School of Medicine, University of Pennsylvania, Philadelphia, PA USA; 5 Department of Pathology, Stanford University, Stanford, CA USA; 6 Department of Bioengineering, Stanford University, Stanford, CA USA; 7 Gladstone–UCSF Institute of Genomic Immunology, San Francisco, CA USA; 8 Center for Personal Dynamic Regulomes, Stanford University, Stanford, CA USA; 9 Department of Genetics, Stanford University, Stanford, CA USA; 10 Department of Microbiology, Perelman School of Medicine, University of Pennsylvania, Philadelphia, PA USA; 11 Department of Pathology and Laboratory Medicine, Perelman School of Medicine, University of Pennsylvania, Philadelphia, PA USA; 12 Department of Neurosurgery, Perelman School of Medicine, University of Pennsylvania, Philadelphia, PA USA; 13 Center for Childhood Cancer Research, Children’s Hospital of Philadelphia, Philadelphia, PA USA; 14 Abramson Cancer Center, Perelman School of Medicine, University of Pennsylvania, Philadelphia, PA USA; 15 Parker Institute for Cancer Immunotherapy, San Francisco, CA USA; 16 Department of Pediatrics, Stanford University, Stanford, CA USA; 17 Department of Medicine, Stanford University, Stanford, CA USA; 18 Present Address: Genentech, South San Francisco, CA USA

**Keywords:** Cancer immunotherapy, Immunotherapy

Correction to: *Nature* 10.1038/s41586-024-07300-8 Published online 10 April 2024

In the version of the article initially published, in the black violin plots in Fig. 5a, a dashed line was used to represent the mean and dotted lines for quartiles. This has now been corrected to a solid line for the mean and dashed lines for the quartiles in the HTML and PDF versions of the article.

