# Supplementary material for: FOXO1 is a master regulator of memory programming in CAR T cells
Source: Nature. 2024 Apr 10;629(8010):211–8. doi: 10.1038/s41586-024-07300-8 (PMC11062920; doi:10.1038/s41586-024-07300-8)
Supplement: Supplementary file 1 — This file includes Supplementary Fig. 1 - the representative flow cytometry gating strategy, Supplementary Fig. 2 - bulk ATAC-seq quality control metrics and Supplementary Fig. 3 - raw western blot data corresponding to Extended Data Figs 6b and 8a. [file 41586_2024_7300_MOESM1_ESM.pdf]

nature portfolio
https://doi.org/10.1038/s41586-024-07300-8

Supplementary information

FOXO1 is a master regulator of memory programming in CAR T cells

In the format provided by the authors and unedited

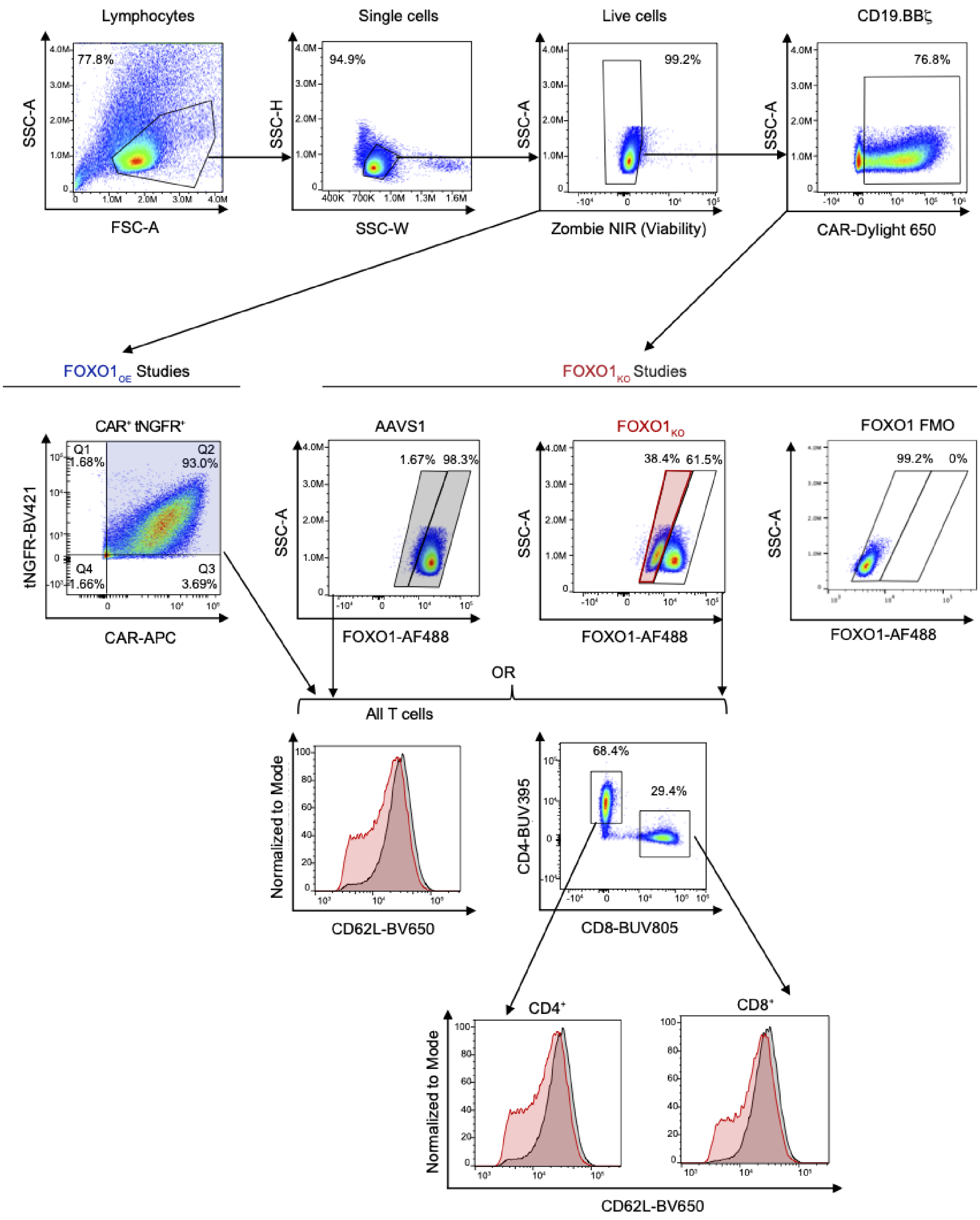

Supplementary Figure 1: Representative flow cytometry gating strategy. Unless otherwise noted, cells in overexpression studies are gated on CAR$^+$ tNGFR$^+$ cells prior to downstream analyses. Cells in *FOXO1* knockout studies are gated on the FOXO1$_{lo}$ population in the FOXO1$_{KO}$ group and on all FOXO1-stained cells in the control AAVS1 control group.

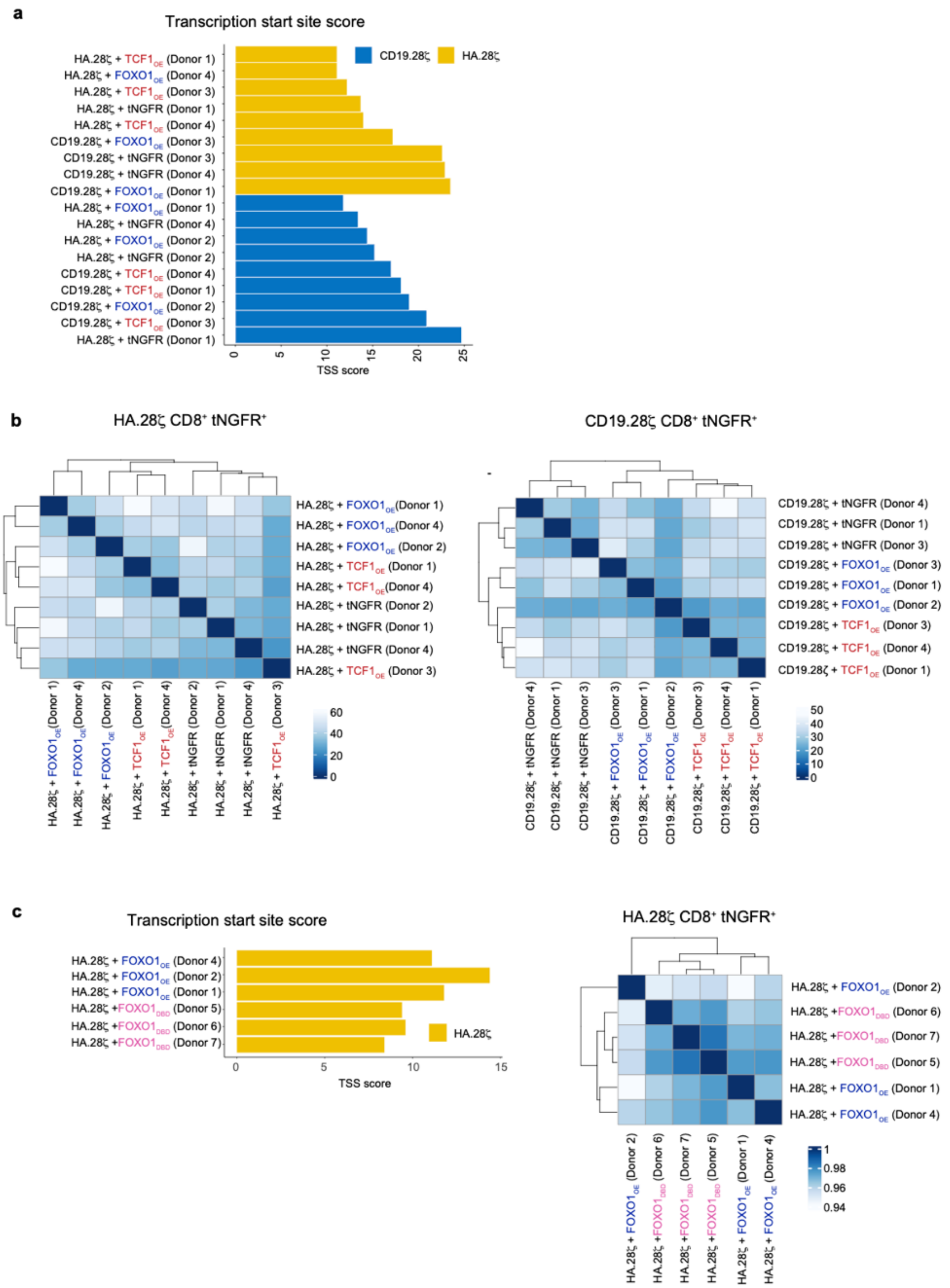

Supplementary Figure 2: Bulk ATAC-seq quality control metrics. a, Transcriptional start site (TSS) enrichment scores for all samples in Fig. 2 and Extended Data Fig. 5. **b,** Pearson correlation and hierarchical clustering of ATAC-sequencing data in Fig. 2 and Extended Data Fig. 5. **c,** TSS enrichment scores and Pearson correlation and hierarchical clustering of FOXO1$_{OE}$ and FOXO1$_{DBD}$ ATAC-seq samples in Extended Data Fig. 8d.

Extended Data Figure 6b

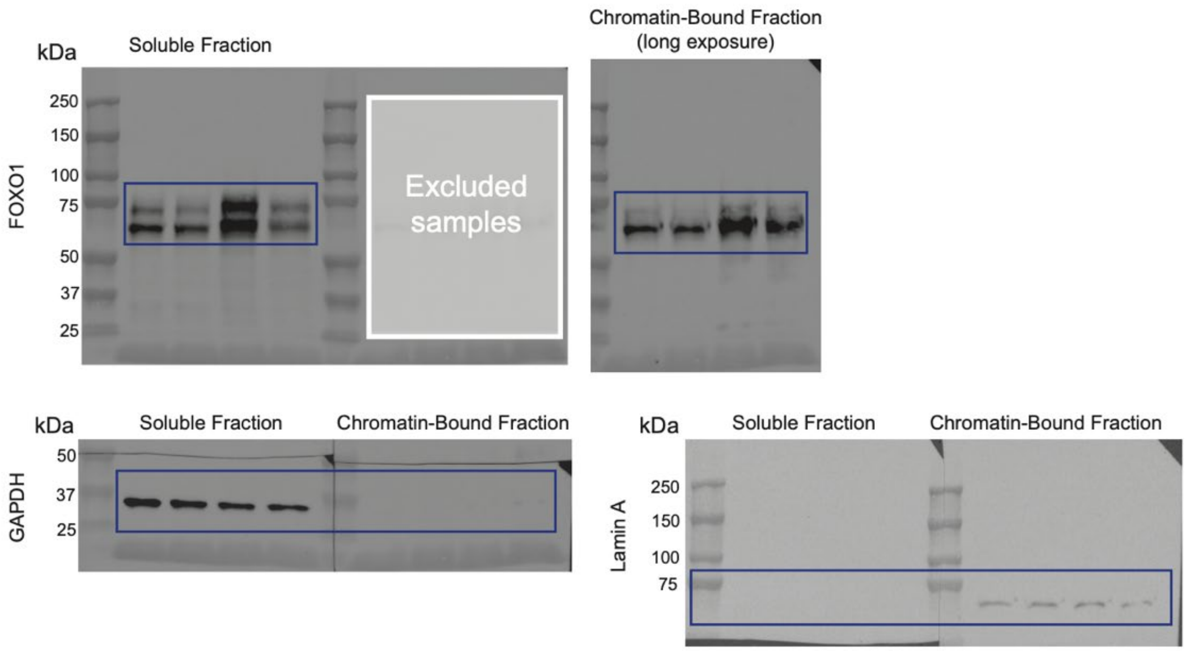

Extended Data Figure 8a

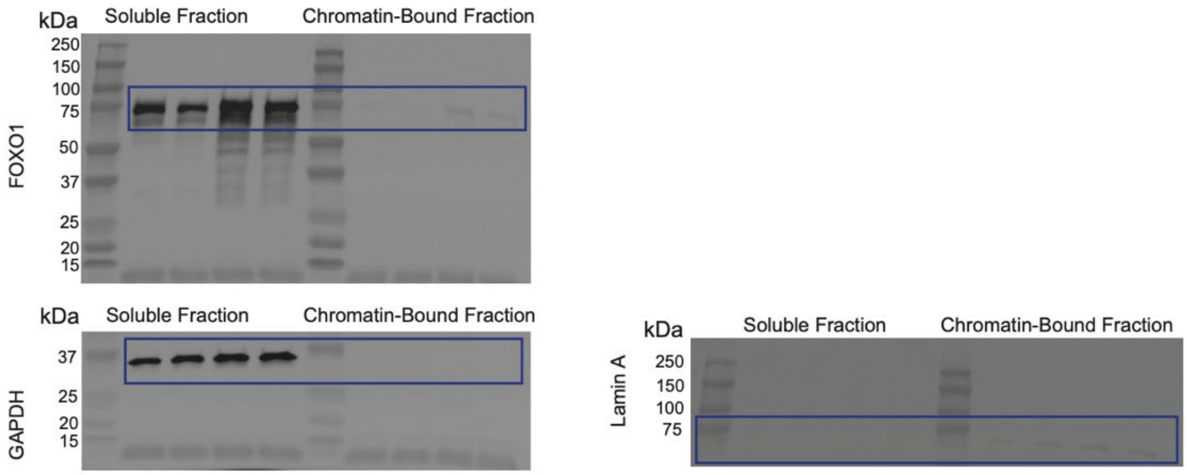

Supplementary Figure 3: Raw western blot data corresponding to Extended Data Figures 6b and 8a. Chemiluminescence and brightfield images were acquired in parallel. Uncropped western blots images are overlaid with a brightfield image of the corresponding ladder from the same gel, and include molecular weight markers. Blots correspond to the data shown in Extended Data Figs. 6b and 8a. Samples that are unrelated to this study or were excluded due to suboptimal signal are indicated by a transparent text box.
