# Supplementary material for: FOXO1 is a master regulator of memory programming in CAR T cells
Source: Nature. 2024 Apr 10;629(8010):211–8. doi: 10.1038/s41586-024-07300-8 (PMC11062920; doi:10.1038/s41586-024-07300-8)
Supplement: Supplementary file 2 — Reporting Summary [file 41586_2024_7300_MOESM2_ESM.pdf]

nature portfolio

Corresponding author(s): Crystal L. Mackall, Evan W. Weber

Last updated by author(s): 02/09/2024

Reporting Summary

Nature Portfolio wishes to improve the reproducibility of the work that we publish. This form provides structure for consistency and transparency in reporting. For further information on Nature Portfolio policies, see our Editorial Policies and the Editorial Policy Checklist.

Statistics

For all statistical analyses, confirm that the following items are present in the figure legend, table legend, main text, or Methods section.

n/a	Confirmed	
☐	☒	The exact sample size (*n*) for each experimental group/condition, given as a discrete number and unit of measurement
☐	☒	A statement on whether measurements were taken from distinct samples or whether the same sample was measured repeatedly
☐	☒	The statistical test(s) used AND whether they are one- or two-sided<br>*Only common tests should be described solely by name; describe more complex techniques in the Methods section.*
☐	☒	A description of all covariates tested
☐	☒	A description of any assumptions or corrections, such as tests of normality and adjustment for multiple comparisons
☐	☒	A full description of the statistical parameters including central tendency (e.g. means) or other basic estimates (e.g. regression coefficient) AND variation (e.g. standard deviation) or associated estimates of uncertainty (e.g. confidence intervals)
☐	☒	For null hypothesis testing, the test statistic (e.g. *F*, *t*, *r*) with confidence intervals, effect sizes, degrees of freedom and *P* value noted<br>*Give P values as exact values whenever suitable.*
☒	☐	For Bayesian analysis, information on the choice of priors and Markov chain Monte Carlo settings
☐	☒	For hierarchical and complex designs, identification of the appropriate level for tests and full reporting of outcomes
☐	☒	Estimates of effect sizes (e.g. Cohen's *d*, Pearson's *r*), indicating how they were calculated

Our web collection on statistics for biologists contains articles on many of the points above.

Software and code

Policy information about availability of computer code

Data collection	Flow cytometry: Data was collected on a BD Fortessa running FACS Diva version 8.0.1 or a Cytek Aurora running SpectroFlo Software version 3.1.0.<br>Cytokine secretion: Data was collected on a Tecan Spark plate reader or a BioTek Synergy H1 running Gen5 version 2.00.18<br>Killing assays: Image collection and analysis was performed on EssenBioscience/Sartorius IncuCyte ZOOM S3 Software<br>Immunoblotting: Image collection was performed on a ChemiDoc using Image Lab Touch Software version 3.0.1.14<br>In vivo analysis: Images were collected and analyzed using Perkin Elmer Living Image version 4.7.3 or Spectral Instruments Imaging Aura version 4.0.7
Data analysis	Figures and Statistical Analysis: Figures were created and statistical tests ere performed on Graphpad Prism version 9.3.1<br>DNA sequence analysis and cloning: DNA sequences were analyzed on SnapGene version 6.0.5<br>Flow cytometry: Flow cytometric data was analyzed FlowJo version 10.8.1<br>Cytokine secretion and killing assays: Data was analyzed on Graphpad Prism version version 9.4.1<br>Seahorse: Data was analyzed on Agilent Seahorse Wave Desktop Software<br>Immunoblotting: Data were using FIJI version 2.14.0/1.5f.<br>RNA-seq: The following analyses were performed on R, version 4.1.0: RNA-seq analysis was performed as per the nf-core RNAseq pipeline version 3.1.1 (https://github.com/nf-core/rnaseq). GSVA scores were calculated via the GSVA pipeline version 1.46.0 (https://github.com/rcastelo/GSVA). Both RNA-seq and ATAC-seq samples were analyzed via DESeq2 version 3.16. Motif search was performed utilizing HOMER version 4.11. Pathway enrichment analyses were performed using clusterProfiler version 4.6.2. Ingenuity Pathway Analysis was performed using QIAGEN Ingenuity Pathway Analysis 2022 Winter Release.<br>ATAC-seq: ATAC-seq analysis was performed as per the PEPATAC pipeline (https://pepatac.databio.org/en/latest/). ATAC enrichment was

performed using gchromVAR (https://github.com/caleblareau/gchromVAR).
Single-cell RNA-seq: FASTQ files were generated and aligned to the genome with Cellranger version 7.1.0. Low quality cells with <300 or >7500 genes or >10% mitochondrial reads were removed using Seurat version 4.3.0. Doublets were identified using DoubletFinder v2.0.3. FindAllMarkers (Seurat) was used to identify differentially expressed (DE) genes in each cluster, and gene ontology (GO) analyses were performed for each cluster using ClusterProfiler version 4.6.2.
FOXO1 Regulon: Regulon analyses on single-cell ATAC-seq data were performed using Seurat v 4.3.0 and GSVA v1.46.0 and gchromVAR.

Code availability statement: All code associated with this paper are deposited to the Weber Lab GitHub (https://github.com/Weber-Lab-CHOP/FOXO1_2024).

For manuscripts utilizing custom algorithms or software that are central to the research but not yet described in published literature, software must be made available to editors and reviewers. We strongly encourage code deposition in a community repository (e.g. GitHub). See the Nature Portfolio guidelines for submitting code & software for further information.

Data

Policy information about availability of data

All manuscripts must include a data availability statement. This statement should provide the following information, where applicable:

- Accession codes, unique identifiers, or web links for publicly available datasets
- A description of any restrictions on data availability
- For clinical datasets or third party data, please ensure that the statement adheres to our policy

Data availability statement: Transcription factor constructs will be made available through Material Transfer Agreements when possible. The bulk RNA-seq, ATAC-seq, and single-cell RNA-seq datasets were aligned to human genome hg38 and have been deposited in the NCBI Gene Expression Omnibus (GEO) and are accessible through the accession number GSE255416.

Research involving human participants, their data, or biological material

Policy information about studies with human participants or human data. See also policy information about sex, gender (identity/presentation), and sexual orientation and race, ethnicity and racism.

Reporting on sex and gender	*Use the terms sex (biological attribute) and gender (shaped by social and cultural circumstances) carefully in order to avoid confusing both terms. Indicate if findings apply to only one sex or gender; describe whether sex and gender were considered in study design; whether sex and/or gender was determined based on self-reporting or assigned and methods used.*<br>*Provide in the source data disaggregated sex and gender data, where this information has been collected, and if consent has been obtained for sharing of individual-level data; provide overall numbers in this Reporting Summary. Please state if this information has not been collected.*<br>*Report sex- and gender-based analyses where performed, justify reasons for lack of sex- and gender-based analysis.*
Reporting on race, ethnicity, or other socially relevant groupings	*Please specify the socially constructed or socially relevant categorization variable(s) used in your manuscript and explain why they were used. Please note that such variables should not be used as proxies for other socially constructed/relevant variables (for example, race/ethnicity should not be used as a proxy for socioeconomic status).*<br>*Provide clear definitions of the relevant terms used, how they were provided (by the participants/respondents, the researchers, or third parties), and the method(s) used to classify people into the different categories (e.g. self-report, census or administrative data, social media data, etc.)*<br>*Please provide details about how you controlled for confounding variables in your analyses.*
Population characteristics	Healthy human donor primary T cells were obtained from the Human Immunology Core at the Perelman School of Medicine at the University of Pennsylvania or from the Stanford Blood Center.
Recruitment	*Describe how participants were recruited. Outline any potential self-selection bias or other biases that may be present and how these are likely to impact results.*
Ethics oversight	*Identify the organization(s) that approved the study protocol.*

Note that full information on the approval of the study protocol must also be provided in the manuscript.

Field-specific reporting

Please select the one below that is the best fit for your research. If you are not sure, read the appropriate sections before making your selection.

☒ Life sciences ☐ Behavioural & social sciences ☐ Ecological, evolutionary & environmental sciences

For a reference copy of the document with all sections, see nature.com/documents/nr-reporting-summary-flat.pdf

Life sciences study design

All studies must disclose on these points even when the disclosure is negative.

Sample size	No sample size calculations were performed; group sizes were validated by experience with well-established, previously published models (1,2).<br><br>1: Lynn, R. C., Weber, E. W., Sotillo, E., Gennert, D., Xu, P., Good, Z., ... & Mackall, C. L. (2019). c-Jun overexpression in CAR T cells induces exhaustion resistance. Nature, 576(7786), 293-300.<br>2: Weber, E. W., Parker, K. R., Sotillo, E., Lynn, R. C., Anbunathan, H., Lattin, J., ... & Mackall, C. L. (2021). Transient rest restores functionality in exhausted CAR-T cells through epigenetic remodeling. Science, 372(6537), eaba1786.
Data exclusions	In the experiment referred to in Figure 5B,C, D, and E, 2 mice in each of the CD19.28ζ tNGFR, CD19.28ζ FOXO1OE, CD19.28ζ FOXO13A, and CD19.28ζ TCF1OE conditions had to be euthanized and data from these mice were excluded due to a non-tumor-related infectious disease complication.
Replication	T cells derived from least 2 different healthy donors were used for each experiment and were tested in a minimum of 2 independent experiments. For experiments where one representative donor was shown, data were representative of all donors. All attempts at replication were successful with the exception of the in vivo experiments noted above in "Data Exclusions" and one CD19.BBζ repeat stimulation experiment as per Figure 2C-F due to extremely low starting numbers of CD8+ T cells in one specific donor that interfered with downstream assays.
Randomization	For in vivo experiments, mice were randomized prior to CAR T cell infusion to ensure equal tumor burden across groups. For other experiments that involved CAR T cell engineering, bulk CD3+ T cells from each healthy donor were randomly distributed into wells prior to viral transduction to ensure equal cellular heterogeneity across groups.
Blinding	In vivo tumor engraftment and T cell infusion were performed by technicians who were blinded to treatments and expected outcomes. Full blinding was not performed for other experiments. Fully-blinded experiments were not possible due to a limited number of investigators capable of performing such experiments.

Reporting for specific materials, systems and methods

We require information from authors about some types of materials, experimental systems and methods used in many studies. Here, indicate whether each material, system or method listed is relevant to your study. If you are not sure if a list item applies to your research, read the appropriate section before selecting a response.

Materials & experimental systems

n/a	Involved in the study	
☐	☒	Antibodies
☐	☒	Eukaryotic cell lines
☒	☐	Palaeontology and archaeology
☐	☒	Animals and other organisms
☒	☐	Clinical data
☒	☐	Dual use research of concern
☒	☐	Plants

Methods

n/a	Involved in the study	
☒	☐	ChIP-seq
☐	☒	Flow cytometry
☒	☐	MRI-based neuroimaging

Antibodies

Antibodies used	For Flow Cytometry:<br>From BD:<br>Anti-CD4 BUV395 (clone: SK3, catalog: 563550); Anti-CD8 BUV805 (clone: SK1, catalog: 612889); Anti-Blimp1 PE-CF594 (clone: 6D3, catalog: 565274); Anti-CD271 BV711 (clone: C40-1457, catalog: 743360); Anti-CD271 BV421 (clone: C40-1457, catalog: 562562); Anti-CD45RA FITC (clone: HI100, catalog: 561882).<br><br>From BioLegend:<br>Anti-CD62L BV605 (clone: DREG-56, catalog: 304834); Anti-CD45RA AF488 (clone: HI100, catalog: 304114); Anti-CD45RA BV711 (clone: HI100, catalog: 304137); Anti-IL7Ra BV421 (clone: A019D5, catalog: 351310); Anti-CD39 BV711 (clone: A1, catalog: 328228); Anti-CD39 APC-Cy7 (clone: A1, catalog: 328225); Anti-TIM3 BV510 (clone: F38-2E2, catalog: 345030); Anti-Tbet BV711 (clone: 4B10, catalog: 644820); Anti-Tbet BV785 (clone: 4B10, catalog: 644835); Anti-CD127 BV711 (clone: A019D5, catalog: 351327); Anti-CD8 AF700 (clone: SK1, catalog: 344723); Anti-CD62L PerCP-Cy5.5 (clone: DREG-56, catalog: 304823)<br><br>From Cell Signaling:<br>Anti-FOXO1 AF488 (clone: C29H4, catalog: 58223S); Anti-TCF1 PE (clone: C63D9, catalog: 14456); Anti-TCF1 AF647 (clone: C63D9,

catalog: 6709S); Anti-Bcl-2 PE (clone: 124, catalog: 26295S); Anti-LEF1 AF488 (clone: C12A5, catalog: 8490S); Anti-LEF1 PE (clone: C12A5, catalog: 14440)

From eBiosciences:
Anti-PD1 PE-Cy7 (clone: J105, catalog: 25-2799-42); Anti-LAG3 PE (clone: 3DS223H, catalog: 12-2239-42)

From Invitrogen:
Anti-CD45 PerCP-Cyanine5.5 (clone: HI30, catalog: 45-0459-42)

Custom antibodies:
Sourced from the National Cancer Institute: Anti-14G2a CAR (clone 1A7, conjugated to Dylight 650 using Thermo Scientific Dylight 650 Labeling Kit catalog #84535)
Sourced from Genscript via custom prep: Anti-CD19 CAR (clone FMC63, conjugated to Dylight 650 using Thermo Scientific Dylight 650 Labeling Kit catalog #84535)

For cell selection:
From BD:
Anti-CD62L PE (clone: DREG-56, catalog: 555544)

From Biolegend:
Anti-CD271 Biotin (clone: ME20.4, catalog: 345122)

For Western Blot:
From Cell Signaling:
Anti-FOXO1 (clone: C29H4, catalog: 2880), Anti-Lamin A (clone 133A2, catalog: 86846), Anti-GAPDH (clone: D4C6R, catalog: 97166), Anti-rabbit IgG, HRP-linked Antibody (clone: n/a, catalog: 7074), Anti-mouse IgG, HRP-linked Antibody (clone: n/a, catalog: 7076)

Validation

All flow cytometry antibodies were validated by manufacturers on various human peripheral blood mononuclear cells except anti-CD271 antibodies which were validated on human neuroblastoma cell line SK-N-MC, which express a high level of NGFR. Antibodieswere additionally validated at Stanford or Children's Hospital of Philadelphia by comparing antibody-specific staining to isotype and unstained controls.
Western blot antibodies were validated by manufacturers on cell lines as noted below in the specific antibody sections.

Antibody validation can be found at the following sites:

Anti-CD4-BUV395: https://www.bdbiosciences.com/en-us/products/reagents/flow-cytometry-reagents/research-reagents/single-color-antibodies-ruo/buv395-mouse-anti-human-cd4.563552
Anti-CD8-BUV805: https://www.bdbiosciences.com/en-us/products/reagents/flow-cytometry-reagents/research-reagents/single-color-antibodies-ruo/buv805-mouse-anti-human-cd8.612889
Anti-CD62L-BV605:
https://www.bdbiosciences.com/en-us/products/reagents/flow-cytometry-reagents/research-reagents/single-color-antibodies-ruo/bv605-mouse-anti-human-cd62l.562719
Anti-Blimp-1-PE-CF594:
https://www.bdbiosciences.com/en-us/products/reagents/flow-cytometry-reagents/research-reagents/single-color-antibodies-ruo/pe-cf594-rat-anti-blimp-1.565274
Anti-CD271-BV711: https://www.bdbiosciences.com/en-us/products/reagents/flow-cytometry-reagents/research-reagents/single-color-antibodies-ruo/bv711-mouse-anti-human-cd271.743360
Anti-CD271-BV421: https://www.bdbiosciences.com/en-us/products/reagents/flow-cytometry-reagents/research-reagents/single-color-antibodies-ruo/bv421-mouse-anti-human-cd271.562562
Anti-CD45RA-FITC:
https://www.bdbiosciences.com/en-us/products/reagents/flow-cytometry-reagents/research-reagents/single-color-antibodies-ruo/fitc-mouse-anti-human-cd45ra.561882
Anti-CD62L-BV605:
https://www.biolegend.com/en-us/products/brilliant-violet-605-anti-human-cd62l-antibody-8554?GroupID=BLG10034
Anti-CD45RA-AF488:
https://www.biolegend.com/en-us/products/alexa-fluor-488-anti-human-cd45ra-antibody-3337?GroupID=GROUP658
Anti-CD45RA-BV711:
https://www.biolegend.com/en-us/products/brilliant-violet-711-anti-human-cd45ra-antibody-7937
Anti-IL-7Ra-BV421:
https://www.biolegend.com/en-us/products/brilliant-violet-421-anti-human-cd127-il-7ralpha-antibody-7155
Anti-CD39-BV711:
https://www.biolegend.com/en-us/products/brilliant-violet-711-anti-human-cd39-antibody-1390
Anti-CD39-APC-Cy7:
https://www.biolegend.com/en-us/products/apc-cyanine7-anti-human-cd39-antibody-12925
Anti-Tim-3-BV510:
https://www.biolegend.com/en-us/products/brilliant-violet-510-anti-human-cd366-tim-3-antibody-12009
Anti-T-bet-BV711:
https://www.biolegend.com/en-us/search-results/brilliant-violet-711-anti-t-bet-antibody-7952?GroupID=BLG6433
Anti-T-Bet-BV785:
https://www.biolegend.com/en-us/products/brilliant-violet-785-anti-t-bet-antibody-15077?GroupID=BLG6433
Anti-IL-7Ra-BV711:
https://www.biolegend.com/en-us/products/brilliant-violet-711-anti-human-cd127-il-7ralpha-antibody-7947?GroupID=BLG9274
Anti-CD8-AF700:

https://www.biolegend.com/en-us/products/alexa-fluor-700-anti-human-cd8-antibody-9062?GroupID=BLG10167
Anti-CD62L-PerCP-Cy5.5
https://www.biolegend.com/en-us/products/percp-cyanine5-5-anti-human-cd62l-antibody-4243?GroupID=BLG10270
Anti-FOXO1-AF488:
https://www.cellsignal.com/products/antibody-conjugates/foxo1-c29h4-rabbit-mab-alexa-fluor-488-conjugate/58223
Anti-TCF1/TCF7-PE:
https://www.cellsignal.com/products/antibody-conjugates/tcf1-tcf7-c63d9-rabbit-mab-pe-conjugate/14456
Anti-TCF1/TCF7-AF647:
https://www.cellsignal.com/products/antibody-conjugates/tcf1-tcf7-c63d9-rabbit-mab-alexa-fluor-647-conjugate/6709
Anti-BCL-2-PE:
https://www.cellsignal.com/products/antibody-conjugates/bcl-2-124-mouse-mab-pe-conjugate/26295
Anti-LEF1-AF488:
https://www.cellsignal.com/products/antibody-conjugates/lef1-c12a5-rabbit-mab-alexa-fluor-488-conjugate/8490
Anti-LEF1-PE:
https://www.cellsignal.com/products/antibody-conjugates/lef1-c12a5-rabbit-mab-pe-conjugate/14440
Anti-PD-1-PE-Cy7:
https://www.thermofisher.com/antibody/product/CD279-PD-1-Antibody-clone-eBioJ105-J105-Monoclonal/25-2799-42
Anti-LAG3-PE:
https://www.thermofisher.com/antibody/product/CD223-LAG-3-Antibody-clone-3DS223H-Monoclonal/12-2239-42
Anti-CD45-PerCP-Cy5.5
https://www.thermofisher.com/antibody/product/CD45-Antibody-clone-HI30-Monoclonal/45-0459-42
Anti-CD62L-PE:
https://www.bdbiosciences.com/en-us/products/reagents/flow-cytometry-reagents/research-reagents/single-color-antibodies-ruo/pe-mouse-anti-human-cd62l.555544
Anti-CD271-Biotin:
https://www.biolegend.com/en-gb/products/biotin-anti-human-cd271-ngfr-antibody-17603
FOXO1 Rabbit mAB: validated on HEK293T cells:
https://www.cellsignal.com/products/primary-antibodies/foxo1-c29h4-rabbit-mab/2880
Lamin A Mouse mAb: validated on HeLa, PC-3, A549, PANC-1, MCF7, and ACHN cells:
https://www.cellsignal.com/products/primary-antibodies/lamin-a-133a2-mouse-mab/86846
GAPDH Mouse mAb: validated on HeLA, NIH/3T3, C6, and COS-7 cells:
https://www.cellsignal.com/products/primary-antibodies/gapdh-d4c6r-mouse-mab/97166
Anti-rabbit IgG, HRP-linked Antibody:
https://www.cellsignal.com/products/secondary-antibodies/anti-rabbit-igg-hrp-linked-antibody/7074
Anti-mouse IgG, HRP-linked Antibody:
https://www.cellsignal.com/products/secondary-antibodies/anti-mouse-igg-hrp-linked-antibody/7076

Eukaryotic cell lines

Policy information about cell lines and Sex and Gender in Research

Cell line source(s)

Nalm6 and 143B cells were obtained from the American Type Culture Collection and engineered as per methods. HEK239GP cells were obtained from the National Cancer Institute. Primary human T cells were obtained from anonymous healthy donor buffy coats via the Stanford University Blood Center under a University Institutional Review Board-exempt protocol or Human Peripheral Blood Leukopaks (StemCell Technologies) at Stanford and from the University of Pennsylvania Human Immunology Core at Children's Hospital of Philadelphia.

Authentication

Nalm6 and 143B cell lines that were engineered to express luciferase and fluorescent proteins (Nalm6-GL and 143B-GL) were verified via flow cytometry. CD19 negative Nalm6 used in tumor re-challenge experiments were verified via flow cytometry. Nalm6 and 143B cell lines and engineered versions of these cell lines were previously authenticated via STR fingerprinting prior to their use in this study.

Mycoplasma contamination

Cells were frequently tested for mycoplasma using the Lonza MycoAlert Mycoplasma Detection kit. All experiments reported in this study used cells that tested negative for Mycoplasma.

Commonly misidentified lines
(See ICLAC register)

None were used.

Animals and other research organisms

Policy information about studies involving animals; ARRIVE guidelines recommended for reporting animal research, and Sex and Gender in Research

Laboratory animals

NOD/SCID/IL2Rγ-/- (NSG) mice were bred, housed, and treated under Stanford University APLAC- or Children's Hospital of Philadelphia (CHOP) ACUP-approved protocols. 6-8 week-old mice were healthy, immunocompromised, drug- and test-naïve, and unused in other procedures. Mice were housed at the Stanford Veterinary Service Center (VSC) or CHOP Department of Veterinary Services (DVR) in a barrier facility with a 12-hour light/dark cycle, and mice were kept at a temperature of 20-23C (CHOP) or 20-26C (Stanford) with humidity ranging from 30-70%.

Wild animals	No wild animals were used in this study
Reporting on sex	Relatively equal numbers of male and female healthy human donor T cells were used for this study. Similarly, in vivo experiments used relatively equal numbers of male and female mice (but were sex-controlled within each individual experiment). Therefore, findings from this study can be applied to both sexes.
Field-collected samples	No field samples were used.
Ethics oversight	All animal studies were undertaken under Stanford University APLAC- or Children's Hospital of Philadelphia (CHOP) ACUP-approved protocols. Mice were monitored daily by VSC or DVR staff and euthanized if endpoint criteria were met.

Note that full information on the approval of the study protocol must also be provided in the manuscript.

Flow Cytometry

Plots

Confirm that:

☒ The axis labels state the marker and fluorochrome used (e.g. CD4-FITC).

☒ The axis scales are clearly visible. Include numbers along axes only for bottom left plot of group (a 'group' is an analysis of identical markers).

☒ All plots are contour plots with outliers or pseudocolor plots.

☒ A numerical value for number of cells or percentage (with statistics) is provided.

Methodology

Sample preparation	For surface phenotyping: as per methods, T cells were washed twice in FACS buffer (PBS + 2% FBS), stained with fluorophore-conjugated antibodies in FACS buffer (100uL total staining volume per sample) for 30 minutes on ice, washed twice again with FACS buffer, and then analyzed.<br><br>For intracellular phenotyping: as per methods, cells were prepared as above with surface stains then fixed, permeabilized, and stained using the eBioscience FoxP3 Transcription Factor Staining Buffer Set as per manufacturer's protocol.<br><br>Cell surface antibodies were used at a 1:100 dilution during staining, with the exception anti-14g2a and anti-FMC63, which were used at a 1:1000 dilution. Intracellular antibodies were used at a 1:50 dilution and live/dead staining was used at a 1:1000 dilution.
Instrument	BD Fortessa (Stanford) and Cytek Aurora (Children's Hospital of Philadelphia)
Software	FACS Diva version 10.8.1 (Stanford) or SpectroFlo (Children's Hospital of Philadelphia)
Cell population abundance	For most phenotyping experiments, between 50,000-500,000 lymphocytes were collected.
Gating strategy	Samples were gated on lymphocytes (FSC-A/SSC-A), single cells (SSC-W/SSC-H), and relevant markers (tNGFR, CAR, CD4, CD8, etc. as specified in the manuscript main text). For cells that were stained with live/dead staining, live cells were also gated into the population of interest (live/dead staining was performed using either Zombie NIR Fixable Viability Kit [Biolegend catalog #423105], Fixable Viability Kit eFluor 506 [eBioscience catalog #65-0866-18], or Fixable Viability Kit eFluor 780 [eBioscience catalog #65-0865-14]).<br><br>For FOXO1 CRISPR KO studies, cells were gated as per above and additionally FOXO1KO cells were gated on the FOXO1 negative subpopulation; AAVS1-edited controls were analyzed regardless of FOXO1 expression.<br><br>For in vivo murine blood analysis, samples were gated on lymphocytes and single cells as above, GFPhi tumor cells were gated out via the FITC channel, Human CD45hi cells were gated in, and CountBright absolute counting beads were used to validate absolute cell numbers.

☒ Tick this box to confirm that a figure exemplifying the gating strategy is provided in the Supplementary Information.
